# Effects of bioirrigation of non-biting midges (Diptera: Chironomidae) on lake sediment respiration

**DOI:** 10.1038/srep27329

**Published:** 2016-06-03

**Authors:** Viktor Baranov, Jörg Lewandowski, Paul Romeijn, Gabriel Singer, Stefan Krause

**Affiliations:** 1 Leibniz-Institute of Freshwater Ecology and Inland Fisheries, Müggelseedamm 310, 12587 Berlin, Germany; 2 Humboldt University of Berlin, Faculty of Mathematics and Natural Sciences, Geography Department, Rudower Chaussee 16, 12489 Berlin, Germany; 3 School of Geography, Earth and Environmental Sciences, University of Birmingham, Edgbaston, Birmingham B15 2TT, UK

## Abstract

Bioirrigation or the transport of fluids into the sediment matrix due to the activities of organisms such as bloodworms (larvae of Diptera, Chironomidae), has substantial impacts on sediment respiration in lakes. However, previous quantifications of bioirrigation impacts of Chironomidae have been limited by technical challenges such as the difficulty to separate faunal and bacterial respiration. This paper describes a novel method based on the bioreactive tracer resazurin for measuring respiration *in-situ* in non-sealed systems with constant oxygen supply. Applying this new method in microcosm experiments revealed that bioirrigation enhanced sediment respiration by up to 2.5 times. The new method is yielding lower oxygen consumption than previously reported, as it is only sensitive to aerobic heterotrophous respiration and not to other processes causing oxygen decrease. Hence it decouples the quantification of respiration of animals and inorganic oxygen consumption from microbe respiration in sediment.

Sediment respiration rates can be influenced substantially by bioturbation^1^,^2^, which encompasses all *“transport processes carried out by animals (and plants) that directly or indirectly affect sediment matrices”*^3^. In particular bioturbation associated ventilation of animal burrows has previously been found to have significant impact on sediment respiration^2^,^4^,^5^. Burrow ventilation involves the rapid exchange of water between the overlying water column and subsurface sediments and is caused by animals flushing their open or blind-ended burrows with overlying water for respiration and feeding purposes^6^. Burrow ventilation induced bioirrigation includes diffusive and advective radial pore water flow that enhances the exchange of solutes between the sediment pore water and the overlying water body^2^,^3^. To date, most studies of lake sediment respiration have not sufficiently accounted for the impact of bioirrigation, even though biogenic sediment reworking and ventilation are assumed to have the potential to increase sediment respiration by a factor of 2–3^6^,^7^,^8^,^9^,^10^. Chironomid (Diptera, Chironomidae) larvae, tubificid worms (Oligochaeta, Tubificidae) and some mayflies (i.e. Ephemeroptera, Ephemeridae)^11^ have been identified as the most important bioturbators in freshwater ecosystems, impacting, for instance, nutrient and carbon turnover^2^ and gas^12^ fluxes across the sediment-water interface (Fig. 1A,B). The advective pumping of water through the burrows of chironomids in their larval stage enhances sediment water exchange and thus, impacting biogeochemical processes at the sediment-water interfaces^2^.

Progress in quantitative understanding of bioirrigation impacts on lake sediment respiration has been somewhat hampered by methodological limitations of measuring sediment respiration^13^,^14^. Most existing methods (sealed cores, benthic flux chambers, respiration chambers, eddy covariance technique) are not capable of distinguishing between respiration of the bioirrigating animals and aerobic bacterial respiration in the sediment^5^,^10^,^15^. The most common approach to determine the contribution of animal respiration to total oxygen consumption is to measure the respiration of animals *ex situ*, such as in respiration chambers^16^,^17^. However, it is known that animals in respiration chambers are stressed, and thus consume more oxygen than when dwelling in the sediment^13^,^17^. This results in an overestimation of faunal respiration in the total sediment oxygen consumption^13^,^17^.

The development of novel reactive tracers offers new approaches for quantifying bioirrigation-stimulated respiration at sediment-water interfaces. The resazurin/resorufin tracer system offers the potential for assessing sediment respiration *in situ* and in real time^18^,^19^,^20^. Resazurin (7-Hydroxy-3H-phenoxazin-3-one 10-oxide) is a blue bioreactive tracer, irreversibly reducing to the highly fluorescent pink resorufin in presence of respiring living cells^14^. Resorufin is stable for a period of several days but can be reversibly reduced to the transparent dehydroresorufin at longer time scales. Resazurin has been applied frequently for quantifying hyphorheic metabolism in streams and rivers, with microcosm experiments by González-Pinzón *et al*.^18^ revealed a strong correlation (r = 0.986) between resazurin to resorufin turnover and oxygen consumption due to aerobic respiration by bacteria. ATP concentration has been correlated to resazurin turnover in cells with reduction possibly linked to NADH dehydrogenase activity^21^. While some details of the biochemistry of resazurin transformation are still a matter of investigation, it is generally understood that resazurin is primarily reduced by aerobic respiration of heterotrophic aerobes^18^,^22^. Resazurin turnover rates are specific to bacterial strains^16^. This tracer system therefore opens new opportunities for quantifying bioirrigation impacts on respiration at sediment-water interfaces. Specifically, it provides the potential to measure *in situ* respiration in open systems with unrestricted continuous oxygen influx. In addition, resazurin is allowing for decoupled quantification of inorganic oxygen consumption and non-heterotrophous oxygen uptake from aerobic sediment respiration as its turnover is mainly by aerobic sediment respiration^20^.

The aim of this study is to quantify the impacts of bioirrigating chironomid larvae (Diptera, Chironomidae, C*hironomus plumosus* group) on lake sediment respiration by applying the resazurin tracer system in laboratory microcosm experiments. This includes establishing if resazurin turnover rates are affected by faunal respiration. We will furthermore quantify how bioirrigation associated increases in sediment respiration change with variable densities of chironomid larvae.

## Results

### Bioirrigation impacts on sediment microbial metabolic activity

Resazurin turnover in the microcosms was strongly affected by the presence of chironomid larvae (experiment 1). Within the first 24 hours, rapid decreases of resazurin and fast increases of resorufin were observed in bioirrigated microcosms, indicating rapid conversion of resazurin to resorufin. In contrast, uninhabited control microcosms showed a slower resazurin turnover as indicated by a lower turnover rate (Fig. 2A). Overall, resazurin turnover in the bioirrigated microcosms was significantly higher than in the uninhabited control microcosms (ANCOVA, p < 0.05, n = 18). Resazurin turnover rates varied in time in both the non-bioirrigated and the bioirrigated treatments, with a general trend of decreasing turnover rates over the course of the experiment (Fig. 2A).

### Closed sediment cores incubations and oxygen measurements

In order to compare oxygen consumption yielded by resazurin with more traditional methods we have measured oxygen consumption in sealed sediment cores equipped with optical oxygen sensors (Fig. 3A). After 24 hours, the total oxygen consumption in cores with and without animals was compared. Bioirrigated cores had consumed significantly more oxygen than cores without animals (ANCOVA, p < 0.05, n = 6, Fig. 3B). Oxygen consumption in non-bioirrigated cores was steady and linear, while in bioirrigated cores, oxygen consumption slowed down after oxygen saturation dropped to 20%. The average oxygen consumption rate was 15.6 mg O_2_ m^−2^ d^−1^ in non-bioirrigated cores and 29.7 mg O_2_ m^−2^ d^−1^ in bioirrigated cores. In general, oxygen consumption measured in sealed cores was significantly higher than oxygen consumption measured for respective larval densities using resazurin (Table 1).

### Chironomid density effects on microbial metabolic activity

Resazurin turnover was strongly influenced by the density of chironomids (experiment 2). Within the first 24 hours, substantial resazurin turnover was observed in all treatments (Fig. 2B). In the first 24 hours, resazurin turnover rate was positively correlated with larval density (Pearson’s r = 0.84, n = 24). Resazurin turnover rate was approximately 3.1 times higher at the largest larval density (2112 larvae·m^2^, mean = 0.21, SD = 0.19) than in the uninhabited control microcosms (0 larvae·m^2^, mean = 0.07, SD = 0.07). The differences were statistically significant (ANCOVA, p < 0.05). Similar to experiment 1, resazurin turnover rates decreased over the course of the experiment (Fig. 2B).

### Relation of resazurin turnover rate to oxygen consumption

Oxygen concentrations in sealed microcosms with chironomid-free sediments were strongly correlated to resazurin turnover rate, with r = 0.93 (Pearson’s correlation, n = 16, p < 0.05) (Fig. 4A) and yielded a conversion factor of 0.13 to translate resazurin turnover rate into oxygen consumption (see equation [5] and methods for details). In the uninhabited control microcoms containing only lake water (no sediment), no resazurin turnover was detected within 48 hours. Little resazurin turnover was observed in sealed respiration chambers with chironomid larvae in U-shaped plastic tubes^17^ but without sediment (Fig. 4B,C). No significant correlation between resazurin turnover rate and oxygen consumption rate was observed (r = 0.21, n = 20, p > 0.05) (Fig. 4B) in this set-up. In the sealed respiration chambers with U-shaped plastic tubes microcosms, chironomid respiration ranged between 0.02–0.27 mg O_2_ h^−1^ larvae^−1^, the average consumption at room temperature (24 °C) was 0.0625 mg O_2_ h^−1^ larvae^−1^. As chironomid respiration was only weakly correlated with resazurin turnover, we used the above calculated conversion factor of 0.13 to estimate oxygen consumption rates (excluding larval respiration) in experimental microcosms with different larval densities (Table 1).

## Discussion

Resazurin turnover (Fig. 2A,B) differed significantly between bioirrigated (inhabited) and non-bioirrigated (uninhabited) sediments investigated in this study. The observed differences in resazurin turnover indicate that the activities of chironomid larvae, whose respiration per se caused no significant resazurin turnover, increased microbial respiration rates in sediments by up to 3 times (Fig. 2A,B). These increases can be attributed to bacterial aerobic respiration since previous research indicated that resazurin turnover rates were not affected by other oxygen-consuming redox processes^18^,^21^,^23^. According to control bottles with lake water only, resazurin also proved to be stable in the water column over the duration of the experiment, which contrasts some previously reported results^19^. Thus, it can be assumed that the entirety of observed resazurin turnover can be attributed to aerobic microbial sediment respiration.

The findings of the present study corroborate other published research that indicated considerable increases (2–2.5 times in our experiments) of respiration in bioirrigated sediments^2^,^7^,^10^. For instance, Soster *et al*.^24^ reported sediment respiration to increase by a factor 3.6 when chironomid larvae were present. Other studies reported that bioturbating freshwater macrozoobenthos can be responsible for around 20% enhancement of sediment respiration^1^,^5^. Granelli^10^ reported 17–55% of sediment column respiration enhancement (in comparison to non-bioirrigated sediment) in the presence of chironomid larvae. Svensson and Leonardson^7^ showed that respiration enhancement exceeded the chironomids own respiration by 2.4 times. Our measurements of chironomid respiration are also consistent with those reported in the literature for *C. plumosus* larvae of similar size and at the same temperature^11^,^12^,^18^. According to these previously published results, 4^th^ instar larvae of *C. plumosus* consume 6–8 μg oxygen h^−1^ mg^−1^ of ash free dry weight (AFDW) at 20 °C. In our experiments larvae were consuming on average 6.26 μg O_2_·h^−1^·mg AFDW.

The resazurin method yielded lower oxygen consumption in open systems compared to the total oxygen consumption in sealed incubation cores. In the absence of *C. plumosus*, oxygen consumption measured with resazurin in open cores was 37% lower than oxygen consumption measured with traditional methods in sealed cores. In bioirrigated cores, resazurin yielded oxygen consumptions 31% lower than the traditional approach with sealed incubation cores. It is unlikely that animal respiration is the main reason for the differences between the resazurin and the traditional approaches because differences were similar in the presence and absence of *C. plumosus*. Other possible sources of oxygen consumption unaccounted by resazurin are inorganic reactions (i.e pyrite formation) or chemoautotrophic aerobic oxidation, anammox etc.^13^.

Both resazurin-based and traditional incubation methods have shown that oxygen consumption in bioirrigated cores (528 larvae·m^−2^) was three times that of uninhabited control cores (Fig. 2). Thus, regardless of the method used, the measured impact of bioirrigation remained the same. Resazurin, however, provided more direct insights into the impact of bioirrigation on aerobic respiration in the sediment. It is advisable to couple resazurin measurements with traditional approaches (i.e core incubations) in order to distinguish between different contributions to total oxygen consumption, i.e. animal’s respiration, bacterial aerobic heterotrophic respiration, photorespiration, Mehler reaction, inorganic oxygen uptake etc.^13^.

Resazurin turnover (and thus, aerobic respiration of the system) was positively correlated with larval density (Fig. 2D, Table 1). However, this relationship was non-linear and saturation of the respiration enhancement curve occurred at higher chironomid densities, translating into a lower chironomid-induced, additional resazurin turnover when expressed on a per-capita basis. Resazurin turnover at maximum larval density (>2000 larvae·m^−2^) was very similar to that measured at 25% and 50% of the maximum larval density (Fig. 2D). This observation strongly suggests a density-dependent suppression of chironomid activities and their impacts on their surroundings. This is in accordance with Aller’s transport-reaction model^25^. He showed, that the spacing of burrows is important for biogeochemical processes at sediment-water interfaces. At higher chironomid densities there might be an overlap of oxidized zones around the burrows, i.e. on a per capita basis the oxidized sediment volume, and thus oxygen consumption and resazurin turnover decrease. If there is a further increase of chironomid densities there is no longer enough space for the chironomids to build their burrows. They will build much shorter burrows or none at all. The bioirrigated sediment volume will drastically decrease, not only on a per capita basis but also in absolute numbers. Metabolic suppression might be also caused by food limitations in densely populated sediments^26^.

No strong correlation (n = 20, r = 0.21) between respiration of chironomids themselves and resazurin turnover was observed in our experiments with *C. plumosus* placed in water-filled microcosms. A possible reason for the negligible direct impact of chironomids on resazurin turnover is the missing direct contact between resazurin dissolved in water and the larvae’s respiration organs. Chironomidae are apneustic insects, which means there are no stigma openings of the tracheal system to the environment^4^. Thus, while gaseous oxygen can diffuse through the cuticle and into tracheas, dissolved resazurin cannot be incorporated into the intracellular respiration chain reactions. The enhancement of respiration observed in the bioirrigated microcosms during our experiments can thus be solely attributed to the bioirrigation-facilitated stimulation of sediment bacterial activity without any influence by the chironomids’ own metabolism.

Although resazurin turnover was recorded in the microcosms with U-shaped plastic tubes, with larvae and water, it was not correlated with the amount of oxygen consumed in the system. This means that only a small amount of the consumed oxygen can be attributed to the processes available for resazurin reduction such as bacterial respiration. Some resazurin turnover, which we have observed, might have been caused by the microbiota, directly associated with the larvae^27^.

The present microcosm study revealed a strong positive correlation (r = 0.93) between oxygen consumption and resazurin turnover rate. These results are in accordance with relationships found previously^18^, yet the here identified correlations were slightly weaker than the ones reported for instance in González-Pinzón *et al*.^18^ (r = 0.986). We attribute this difference to the fact that González-Pinzón *et al*.^18^ used pure bacterial cultures whilst the present study applied sediment hosting complex bacterial communities and meiofauna below the sieving threshold of the applied defaunation procedure. In this respect the experimental setup of the present study represents more realistic (natural) sediment conditions, with the consequence that inorganic O_2_-consuming reactions may have caused minor perturbations of the oxygen signal, and thus, marginally reduced the strength of the correlation of r = 0.93 with the resazurin turnover as compared to pure bacterial communities in González-Pinzón *et al*.^18^.

**Figure 1:**
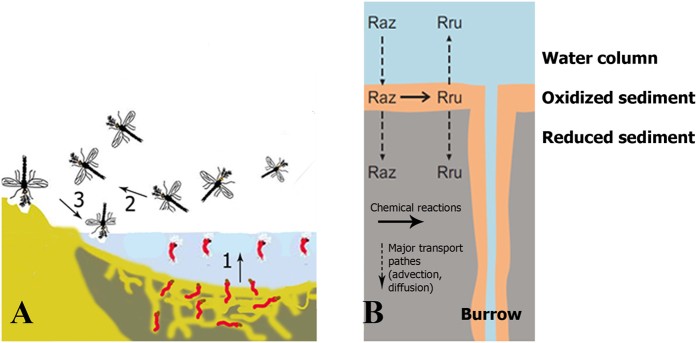
(**A**) Role of chironomids (Diptera, Chironomidae) in biogeochemical processes in lake sediments. At the different stages of their life cycle, bioturbating chironomid species such as the wide spread *Chironomus plumosus* L., 1758, are engaged in the cycling of various chemical elements in aquatic and terrestrial ecosystems: 1. Larvae of chironomids are enhancing fluxes of oxygen, ammonia, phosphorous across the sediment-water interface, by forced sediment ventilation, nutrient excretion, promotion of both inorganic reactions and bacterial activity. 2. Flying adults transfer organic matter and various elements from aquatic to terrestrial ecosystems. 3. Parts of the dead adults are usually flushed back to the water providing an additional route for organic matter flux to aquatic ecosystems. (**B**) Conceptual model of resazurin turnover in bioirrigated sediments. Solid arrows indicate irreversible chemical reactions of the Raz/Rru tracer system, which occur only in oxidized sediments (burrow walls and sediment surface). Dashed arrows indicate diffusive and advective transport between the three compartments shown in different colors.

The fact that – for chironomids – the resazurin tracer system enables a direct assessment of bioirrigation impacts on bacterial respiration in sediments is particularly intriguing when considering that classic measurements based on oxygen consumption largely fail to separate the respiration impacts of chironomids (or other bioirrigators) and sediment microbial communities. Differentiation of these pathways previously relied on separate measurements of animal respiration in sediment-free respiration chambers^16^,^17^. Such measurements, however, are prone to artifacts caused by animal stress^17^. Further, classical oxygen-based assessments can be confounded by inorganic reactions consuming oxygen^4^,^5^,^10^,^11^ which may be facilitated by bioirrigation but are not directly associated to microbial metabolic activity. The conceptual model of bioirrigation impacts on resazurin turnover at the base of this study (Fig. 1B) as well as previous findings with the resazurin tracer system suggest that it likely remains unaffected by non-heterotrophic respiration related redox reactions^18^,^21^. Chironomid activity facilitates diffusion of resazurin into the sediment pore water – alongside with oxygen and probably other nutrients. Within the sediment, increased microbial metabolism causes resazurin turnover, the produced resorufin has to be transported back into the burrow and the main water by diffusion and advection. In a given system (such as an experimental column or a defined surficial sediment layer), chironomid activity causes a larger fraction of sediment volume to be oxygenated and be involved in resazurin turnover^2^.

The application of resazurin as a tracer in microcosm bioturbation experiments revealed several technological challenges. Resazurin and resorufin fluorescence analysis requires low-turbid water because high turbidity or sediment resuspension could affect the fluorescence measurements^28^. Resuspension of organic-rich detritus could furthermore cause abnormally high turnover of resazurin in the water column which would lead to a possible overestimation of system respiration. In order to avoid interference of the results with turbidity and sediment resuspension in the microcosms’ experiments, water should be replaced after initial microcosm filling, in order to remove initial turbidity. Aeration stones or tubes should be placed in the microcosms with great care in order to avoid sediment resuspension.

**Figure 2:**
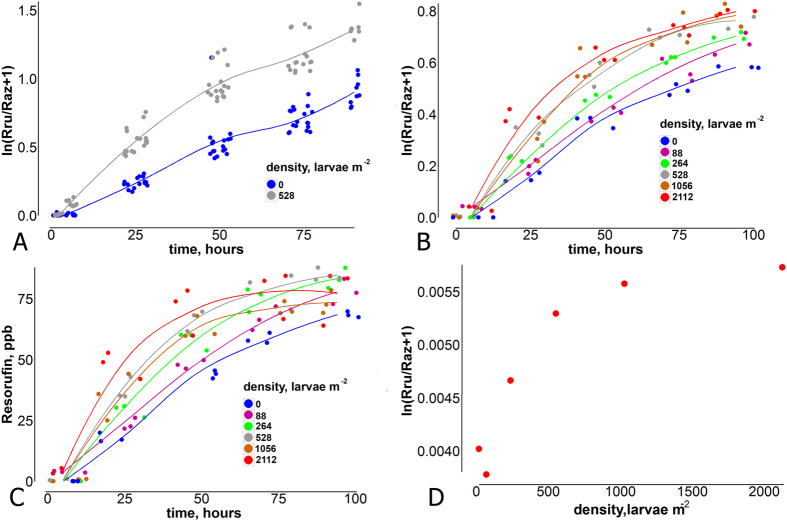
(**A**) Resazurin turnover rate (ln(Rru/Raz + 1) in Experiment 1 with bioirrigated versus non-bioirrigated sediment. Loess smoothing is used to draw a lines here and below. (**B**) Resazurin turnover rate in Experiment 2 with different densities of Chironomidae bioirrigating the sediment. (**C**) Resorufin accumulation over time in Experiment 2. (**D**). Resazurin turnover rates of sediments with different larval densities.

This substantial non-linearity (Fig. 2A,B) in resazurin turnover rate is in strong contrast to previous studies^14^,^18^,^29^. Resazurin turnover rates increased fastest during the first 24–48 hours (Fig. 2B). After 48 hour (depending on animal density) resazurin turnover rates showed non-linear saturation tendencies in all microcosms which was more pronounced in the microcosms with higher chironomid densities. It is likely that this non-linearity is due to the longer duration of this study’s experiments, as compared to previously published stream tracer tests, which normally lasted for several hours only^14^,^18^.

We think several different reasons account for this non-linearity of resazurin turnover: The transformation of resorufin to the colorless compound dehydroresorufin decreases the concentration of the reaction product resorufin in both the overlying and the pore water^18^,^21^. The apparent decrease of resorufin concentration in microcosms with high larval density towards the end of the experiment supports this assumption (Fig. 2C). A sorption of resazurin and resorufin to the sediment matrix may alter the concentrations of both compounds in the overlying water. It was assumed that resazurin and resorufin may have different sorption rates at some conditions, which are sediment and pH specific^18^. Suggested unequal sorption may be one of the reasons why it has been impossible so far to close the resazurin mass balance so far. Nevertheless, the majority of the studies reports equal sorption of both compounds, i.e. we assume that equal sorption is a reliable assumption^18^,^19^,^30^. Diffusion and advection of surface water into the pore water pool (which in our case is about 30% of the volume of the overlying water) which is stored in the sediment compartment alter the concentrations of both resazurin and resorufin in the overlying water. Chironomid-induced bioirrigation promotes advective transport of resazurin into the sediment, i.e. non-linearity in resazurin turnover rate might be partly caused by the difference in advective transport due to chironomid pumping^31^. Brand *et al*.^31^ showed that chironomid bioirrigation creates considerable advective flow (7 × 10^−6^ m s^−1^) into the burrow wall for sediments of comparable hydrological conductivity, resulting in substantial tracer loss from the overlying water column. In case that sorption and advection would be the two main mechanisms regulating resazurin decrease in the water column, one would expect lowest resazurin and resorufin concentrations in the tanks with highest animal densities due to highest advection and sorption. On the contrary, in our experiments resorufin concentrations were increasing directly proportional to the animal density (Fig. 2C). It is remarkable that not only higher resorufin concentrations were observed in the tanks with higher animal densities, but also resorufin concentrations were showing some decrease in the microcosms with 1056, 2112 animals m^−2^ (Fig. 2D). We assume that this might be caused by dehydroresorufin formation. Thus, we conclude that respiration is the main process affecting resazurin turnover in our system.

This study hence provides compelling evidence of the importance of bioturbation effects for sediment metabolism and demonstrates the suitability of the resazurin tracer as a new quantitative method that extends the capabilities of existing technologies for measuring sediment respiration.

## Methods

### Sediment and organisms

Sediments for microcosm experiments were collected from Langer See, a shallow eutrophic lake in North-East Germany (N 52.244592, E 13.787108), on two occasions (February 2014, December 2014). The upper 20 cm of the sediment (key characteristics in Table 2) were collected with an Ekman benthic grab sampler at a distance of approximately 150 m from the lake shore at 4 m water depth. The sediment was homogenized with an electric stirrer and sieved (250 μm) for complete defaunation prior to placement in microcosms.

*Chironomus* gr. *plumosus* sensu Orendt *et al*.^32^ from the Langer See and commercially available sources (Manhard Aquaristik, Berlin) have been used for the experiments. The latter were identified based on rearing of adults as *C*. gr. *plumosus* sensu Orendt *et al*.^32^ and *C*. gr*. salinarius* sensu Orendt *et al*.^32^. Before chironomids for the experiments were handled as described in^4^, larvae were kept in sediment at 4 °C and acclimatized to microcosm conditions for five days prior to transfer into microcosms. Prior to randomly distributing animals across microcosms they were sorted to the same larval stage (4^th^ stage, size 19–25 mm). Initial experiments were performed with both species (“wild” and supplied *C. plumosus* and *C. salinarius*) and confirmed that the choice of species did not influence experimental outcomes. Manipulations were non-invasive and not harmful for animals, and were performed in compliance with German and international laws and ethical guidelines.

### Experimental setup

A first setup (from here called “experiment 1”, Fig. 5A) compared defaunated sediment with no chironomid larvae (“control”, n = 9) to microcosms inhabited by 6 larvae, equivalent to an abundance of 528 specimens per m^2^ (n = 9). In a second setup (from here called “experiment 2”, Fig. 5B), resazurin turnover rates were investigated as a function of chironomid density. For this six microcosm treatments were set up (n = 3 each), including additions of different numbers of chironomid larvae: 0, 1, 3, 6, 12, 24 larvae per microcosm representing the equivalent of 0, 88, 264, 528, 1056 and 2112 specimens per m^2^, respectively (Fig. 5B).

### Microcosm design and preparation

Cylindrical glass microcosms (630 ml, cross-sectional area 105 cm^2^, Fig. 5C) were filled with 300 g of wet sediment and 250 ml of tap water (bank-filtrated water from Großer Müggelsee - shallow lake within Berlin city limit). Porewater volume in each microcosm was estimated to be 270 ml based on established sediment porosities, and a sediment water content of 89% (see Table 2). Water was added carefully on top of the sediment to minimize sediment resuspension. Experiments were conducted in a climate chamber under constant temperature (20° C) and in darkness to prevent oxygen production by benthic algae as well as photodegradation of resazurin^22^. In all microcosms water was constantly aerated by pressurized air to assure permanent oxygen saturation, which was continuously monitored with an oxygen multiprobe (Multi-3430). 72 hours after the initial setup, 80% of the water in each microcosm was removed by a peristaltic pump and replaced by fresh water to reduce initial turbidity to reduce potential interference with the resazurin fluorescence measurements. Chironomids were added to the microcosms a further 48 hours after water replacement. 72 hours afterwards, resazurin was injected into the overlying water and equally distributed in the water column by gentle stirring.

### Quantification of sediment microbial metabolic activity

Sediment microbial metabolic activity was analyzed using of the resazurin/resorufin tracer system^14^. Resazurin stock solutions were prepared for both experiments separately by dissolving resazurin sodium salt (dye content ~80%) in deionized water, producing a resazurin stock solution with a concentration of 1000 ppb. Resazurin was added to microcosms by replacing 20 ml of water with the stock solution results in a dilution of 1 magnitude (estimates of resazurin turnover were normalized accounting for minor variability in starting concentrations)^30^. Based on the measured tracer concentration after mixing overlying water and tracer solution the overlying water volume were calculated. Samples were extracted by 25 ml syringe and analyzed twice a day (10 am and 17 pm) in experiment 1 and once a day (10 am) in experiment 2, for a period of five days. Measurements of resazurin and resorufin fluorescence were conducted immediately after sampling with an Albilla GGUN-FL 30 fluorimeter^28^. After measurements the water samples were transferred from the fluorometer chamber back into the respective microcosm.

From measured resazurin and resorufin concentrations, normalized **resazurin turnover** (as ratio between resorufin and resazurin at the given moment of time) was computed according to Haggerty (2013)^30^ as:


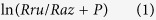


where Rru and Raz are concentrations of resorufin and resazurin in the microcosm at any given moment in time, and P is the production-decay ratio of resorufin. P is assumed to be 1 since the amount of resazurin reduced to resorufin is equal to 1 as the amount of resorufin transformed to dehydroresorufin is negligible on sufficiently short time scales. The slope of the line produced by linear regression of the above mentioned resazurin turnover over time:


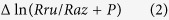


(**resazurin turnover rate**) has been widely accepted as a good indicator of aerobic respiration^14^,^18^,^21^,^30^. Resazurin turnover rates of different larval densities microcosms have been used to analyze the chironomid density impact on sediment respiration. To ensure results are not affected by potential early onset of resorufin breakdown into dehydroresorufin, only the first 48 hours of measurements were used in the data analyses of this study. This approach also seemed feasible in the face of the obvious non-linearity identified for the relationship between resazurin turnover at times >48 h. To test for differences in resazurin turnover between treatments, ANCOVA was applied with ln(Rru/Raz + 1) as the response, time as covariate and the interaction between treatment and time to identify heterogeneity of turnover rates. In experiment 2, resazurin turnover within the first 48 hours were further analyzed as a function of larval densities (Fig. 2D).

### Closed sediment cores incubations and oxygen measurements

To compare resazurin-yielded results with traditional method for measuring oxygen consumption, we have incubated six sealed sediment cores with and without animals for 24 hours. These measurements were taken parallel to “experiment 1” trials, and we used animals and sediment from the same stock.

**Figure 3:**
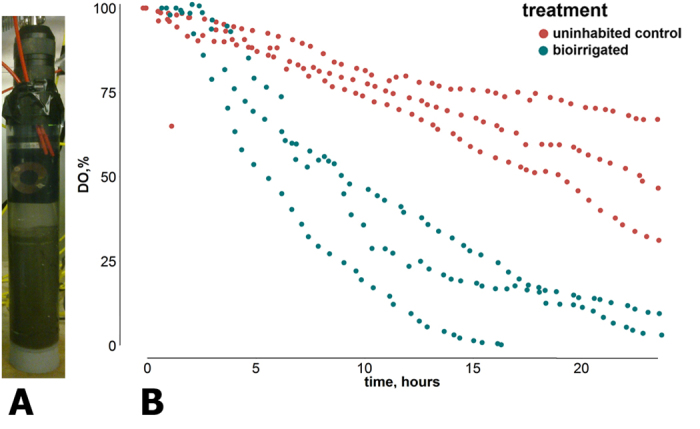
(**A**) Sealed columns, equipped with optical oxygen loggers to measure oxygen consumption in the course of the experiment. (**B**) Oxygen consumption in uninhabited control (0 larvae·m^2^) and bioirrigated (528 larvae·m^2^) sealed columns.

Perspex columns with an inner diameter of 5.8 cm and a sediment surface area of 105 cm^2^ were used. Columns were higher than glass columns from the main experiment in order to accommodate the loggers. Each column contains 300 g of the defaunated (see above) sediment of Langer See and 250 ml of water. Three columns were uninhabited control cores, the other three contained six larvae per column (density equivalent to abundance of 528 specimens per m^2^). Oxygen consumption was measured using optical oxygen loggers Zebra Opto-D installed at the top of each water column. The entire systems were sealed airtight (Fig. 3A). To mix the water during the incubation a peristaltic pump was used. Water was pumped through a tube with 0.5 cm diameter from the water body to the pump and back into the water column at a pumping rate of 5 mL/min. The same peristaltic pump was used to fill the columns with water at the beginning of the experiment (Fig. 3A). Columns were filled to the rim in order to avoid re-aeration. After 24 hours columns were dismantled and data from loggers collected.

### Quantification of oxygen consumption

**Figure 4:**
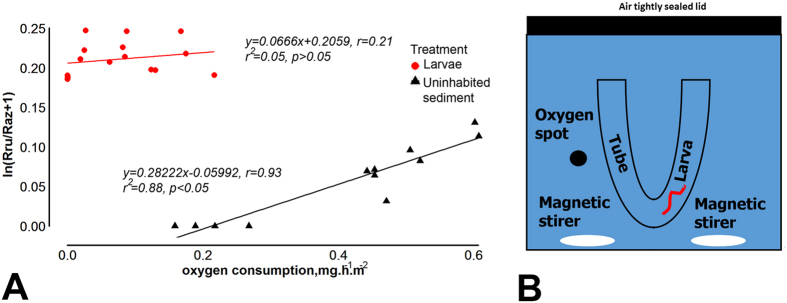
(**A**) Resazurin consumption is well correlated with oxygen consumption in sealed experimental columns with sediment and water only No substantial correlation was found between oxygen consumption by Chironomidae larvae and resazurin turnover rate in the sealed respiration chamber with U-shaped tubes, imitating the chironomid burrow and water. (**B**) Setup in which measurements were conducted.

**Figure 5:**
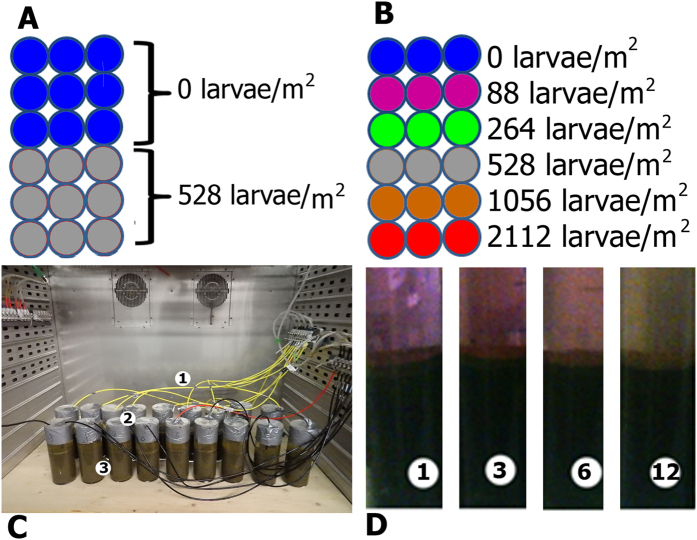
Experimental setup. (**A**) Experiment 1: blue and pink dots represent uninhabited control microcosms (filled with defaunated sediment) and experimental microcosms (with six larvae per microcosm). (**B**) Experiment 2: each color represents one set of 3 microcosms with a same larval density. (**C**) Microcosms installation and setup in the climate chamber: Tubes (1) provide pressurized water-saturated air for constant mixing and aeration. Mesocosms are covered by parafilm (2) to reduce evaporation. Glass cylinders contain water and sediment. (**D**) Development of fluorescent dye tracer in experimental columns with different larval density after 2 weeks of the experiment. Numbers at the bottom of the columns correspond to numbers of larvae in each of the columns. The color differences are due to different resorufin to resazurin ratios.

Resazurin turnover is directly proportional to oxygen consumption rates, yet to translate the tracer measurements to oxygen consumption a system-specific conversion factor has to be applied. Gonzalez Pinzon *et al*.^18^ showed that the resazurin turnover rate (slope of ln(Rru/Raz + 1) over time) to oxygen consumption rate is specific for different sediments and bacterial cultures. Therefore, in this study simultaneous measurements of resazurin turnover and oxygen consumption were carried out in various sealed systems without gas headspace: (i) uninhabited chironomid-free sediment (100 g, Langer See) in closed 250 mL Pyrex bottles filled completely with bank-filtrated lake water of Müggelsee, (ii) chironomid larvae in sediment-free respiration chambers (400 ml Perspex aquaria (Fig. 4C) with u-shaped artificial burrows to reduce animal stress^7^), and (iii) closed 400 mL vials with lake water filtered through 0.2 μm filters. All these experiments were run at shorter time scales and with only modest oxygen consumption to avoid effects of low oxygen concentrations. Bottles and aquaria were equipped with planar optode sensor spots to measure dissolved oxygen non-invasively through the glass wall using fibre-optic technology (Presens Microx 4)^33^. Resorufin concentrations were measured by withdrawing sample volumes of 10 ml with a syringe after 48, 72 and 96 hours. After completion of the measurement each sample was carefully injected back into the microcoms, formation of headspace bubbles was avoided.

The calculation of the resazurin turnover rate ∆(ln(Rru/Raz + 1)) to the oxygen uptake rates


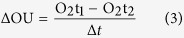


yields the conversion factor *y* in order to compute respiration from resazurin turnover rate:


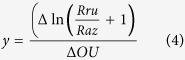


In the above, ∆OU is oxygen consumption rate, O_2_t_1_ - oxygen concentration at time t_1_, O_2_t_2_ - oxygen concentration at time t_2_.

Oxygen consumption rates in columns during the experiment were calculated by equation [4]:


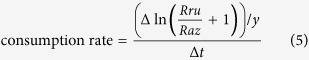


## Additional Information

**How to cite this article**: Baranov, V. *et al*. Effects of bioirrigation of non-biting midges (Diptera: Chironomidae) on lake sediment respiration. *Sci. Rep*. **6**, 27329; doi: 10.1038/srep27329 (2016).

## Figures and Tables

**Table 1:** Oxygen consumption per microcosm in mg O_2_ m^−2^ d^−1^ calculated based on the resazurin turnover rate of the different treatments of experiment 2.

0 larvae m^−2^	88 larvae m^−2^	264 larvae m^−2^	528 larvae m^−2^	1056 larvae m^−2^	2112 larvae m^−2^
9.9	12.7	14.9	20.6	19.4	25.6

**Table 2:** Chemical and physical properties of lake sediments used in the laboratory experiments.

N in %	C in %	S in %	Loss of ignition in %	Water content %
1.3	18.5	2.1	21.1	89

Sediments were collected with an Ekman grab sampler and homogenized with an electric stirrer.
